# Tripterygium Wilfordii inhibits tonsillar IgA production by downregulating IgA class switching in IgA nephropathy

**DOI:** 10.18632/oncotarget.22561

**Published:** 2017-11-20

**Authors:** Huining Li, Dan Kong, Yangyang Xu, Xiaomei Li, Guodong Yao, Kexin Chen, Qi You, Qingtao Shi, Lei Zhang, Xin Wang, Dawei Yuan, Shusheng Miao, Jingshu Geng, Xiaoming Jin, Hongxue Meng

**Affiliations:** ^1^ Department of Pathology, Harbin Medical University, Harbin, China; ^2^ Department of Pathology, Harbin Medical University Cancer Hospital, Harbin, China; ^3^ Department of Pathology, The First Affiliated Hospital of Hei Longjiang University of Chinese Medicine, Harbin, China; ^4^ Department of Gynecology, Harbin Medical University Cancer Hospital, Harbin, China; ^5^ Department of Urinary Surgery, Harbin Medical University Cancer Hospital, Harbin, China; ^6^ Department of Gastroenterology, Harbin Medical University Cancer Hospital, Harbin, China; ^7^ Department of Otolaryngology, Head and Neck Surgery, Second Hospital Affiliated to Harbin Medical University, Harbin, China; ^8^ Department of Otolaryngology, Head and Neck Surgery, Harbin Medical University Cancer Hospital, Harbin, China; ^9^ Geneis (Beijing) Co.Ltd, Beijing, China

**Keywords:** Tripterygium Wilfordii, IgA nephropathy, tonsil, thymic stromal lymphopoietin, IgA class switching

## Abstract

IgA nephropathy (IgAN) is characterized by high serum IgA levels and IgA deposition in the renal mesangium. Recent research has indicated that pathogenic IgA may originate from affected tonsils, where present enhancement of IgA production by IgA class switching and immuno-activation. Tripterygium Wilfordii (TW) was found to be especially effective in IgAN by its’ immunosuppression effect. Given this background, we investigated the mechanisms underlying the role of TW in the generation of IgA and IgA class switching in tonsillar GCs of IgAN patients. Immunohistochemistry and RT-PCR revealed that the expression of thymic stromal lymphopoietin (TSLP) and IgA inducing cytokines were decreased in the tonsils of IgAN patients with TW treatment compared with those without treatment, followed by significantly decreased of IgA-bearing cells. The location of TSLP and IgA inducing cytokines in tonsillar tissue was confirmed by double immunofluorescence. Importantly, TW inhibit TSLP and IgA production in isolated FDC-associated clusters. Serum TSLP levels were decreased and correlated with IgA downregulation in the tonsils and serum of IgAN patients. These data indicated that TW may be involved in IgA production in the tonsils of IgAN patients, inhibiting IgA class switching in IgAN patients through the cooperative roles of AID, TGF-β1, BAFF, and APRIL, highlighting a promising strategy for therapeutic intervention in IgAN.

## INTRODUCTION

Immunoglobulin A nephropathy (IgAN), the most common form of primary glomerulonephritis worldwide, is characterized by qualitative abnormalities in circulating IgA and IgA deposition in the renal mesangium [1, 2]. Pathogenic IgA has been suggested to be crucial to the pathogenesis of IgAN, recent studies have suggested that tonsils are closely related to IgAN and that pathogenic IgA in IgAN is partly of tonsillar origin [3]. Chronic and recurrent tonsillitis are thought to play an important role in new onset and progression of IgAN [4]. Tripterygium Wilfordii (TW), or Lei Gong Teng, also named Thunder God Vine, was found to be especially effective in autoimmune diseases including IgAN, rheumatoid arthritis, psoriasis and lupus by its’ immunosuppression effect [5, 6]. Moreover, the benefits of TW in patients with IgAN suggests that TW may be closely related to tonsillar IgA production. However, the mechanism of TW in tonsillar IgA production in IgAN is unknown.

Palatine tonsils have deep, branched, antigen-retaining crypts with a reticular epithelium, provide a first line of defense against inhaled foreign pathogens. Our previous studies have shown that the number and relative percentage of IgA-bearing cells were significantly increased in the tonsils of IgAN patients [7]. The germinal center (GC) is the main site of B cell proliferation and IgA class switching supported by follicular dendritic cells (FDCs) [8]. Within the primary follicles and germinal centers (GCs), B cells interact functionally with FDCs and undergo critical functional processes, including proliferation, apoptosis, somatic hypermutation, selection for high-affinity antigen binding, isotype switching, and differentiation into plasma cells or memory cells [8, 9].

Upon activation by antigen and accessory signals, tonsillar GC naive IgM^+^ IgD^-^ B cells may acquire IgA expression by undergoing class switch recombination (CSR) [10]. IgA class switching is initiated by production of Iα-Cα germline transcripts (GLTs) and mediated by activation-induced cytidine deaminase (AID), yielding a chimeric Iα-Cμ switch circle transcript [11, 12]. Details of CSR are shown in our previous study [7]. For CSR, tonsillar crypt epithelium is activated to secrete thymic stromal lymphopoietin (TSLP), an interleukin (IL)-7-like type 1 cytokine, which further promotes class switching. Furthermore, IgA switching may rely on proliferation- and survival-inducing cytokines of the tumor necrosis factor (TNF) family, such as B cell-activating factor of the TNF family (BAFF) and a proliferation-inducing ligand (APRIL) secreted by activated DCs and FDCs [10-13]. Transforming growth factor (TGF)-β1 is also involved in IgA switching by promoting germline transcription. By releasing IgA-inducing cytokines (TGF-β1, BAFF, and APRIL), FDCs enhance IgA production in IgAN [14]. However, the involvement of TW in IgA production in tonsillar GCs of patients with IgAN is unknown. Furthermore, the molecular and cellular mechanisms underlying the role of TW in the generation of IgA and IgA class switching in tonsillar GCs of IgAN patients remain largely unknown.

The objective of this study was to investigate the involvement of TW in tonsillar IgA production and to elucidate the molecular basis underlying the role of TW in the generation of IgA and IgA class switching in IgAN. Our study demonstrated that TW may inhibits tonsillar IgA class switching and IgA production by downregulating TSLP and IgA inducing cytokines.

## RESULTS

### IgA-bearing cells was decreased in the tonsils of IgAN patients with TW treatment than in those without treatment

**Figure 1:**
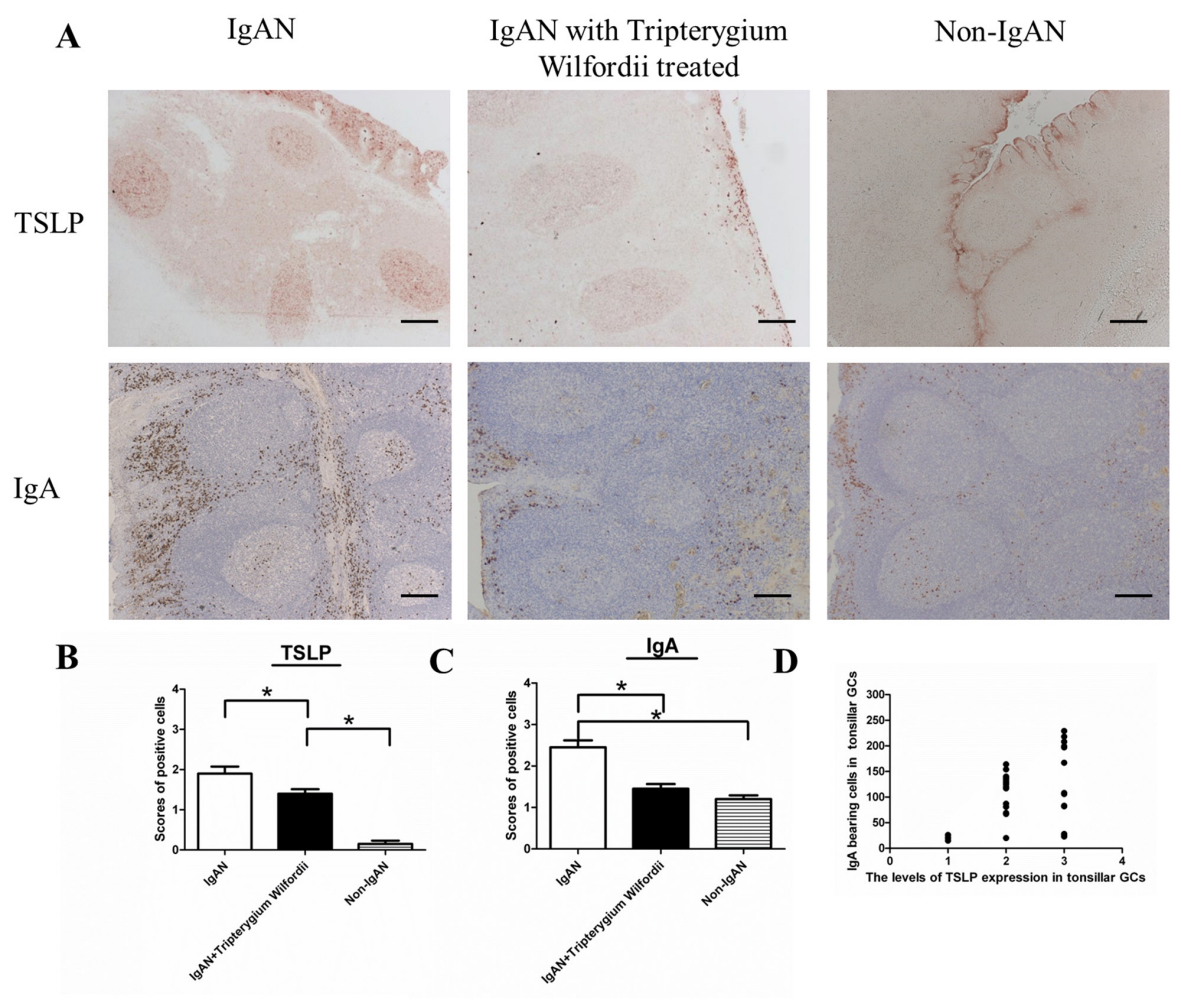
IgAN patients with Tripterygium Wilfordii treatment exhibited decreased numbers of IgA-bearing cells in their tonsils **(A)** Immunohistochemistry of Thymic stromal lymphopoietin (TSLP) and IgA in the tonsils of IgAN patients and non-IgAN patients with chronic tonsillitis showed the presence of TSLP and IgA-bearing cells in the follicular germinal centers (GCs), reticular crypt epithelium (Ep), and subepithelial area. Bars, 200 μm. **(B, C)** The number of TSLP and IgA-bearing cells in the tonsils was counted in 10 randomly chosen, low-power (100× magnification) fields for each patient. The slides were analyzed in blinded manner by two independent investigators. n = 20 for IgAN patients with Tripterygium Wilfordii treatment, n = 20 for IgAN patients without treatment and n = 20 for non-IgAN patients with chronic tonsillitis. Error bars indicate SEMs. ^*^, *P* < 0.01 (Mann-Whitney U test). **(D)** The Y axis label on the graph (third row) showed “the number of IgA bearing cells in tonsillar GCs”, and the X axis label showed “the levels of TSLP expression in tonsillar GCs”. Correlation between IgA and TSLP was analyzed by Spearman’s correlation.

**Figure 2:**
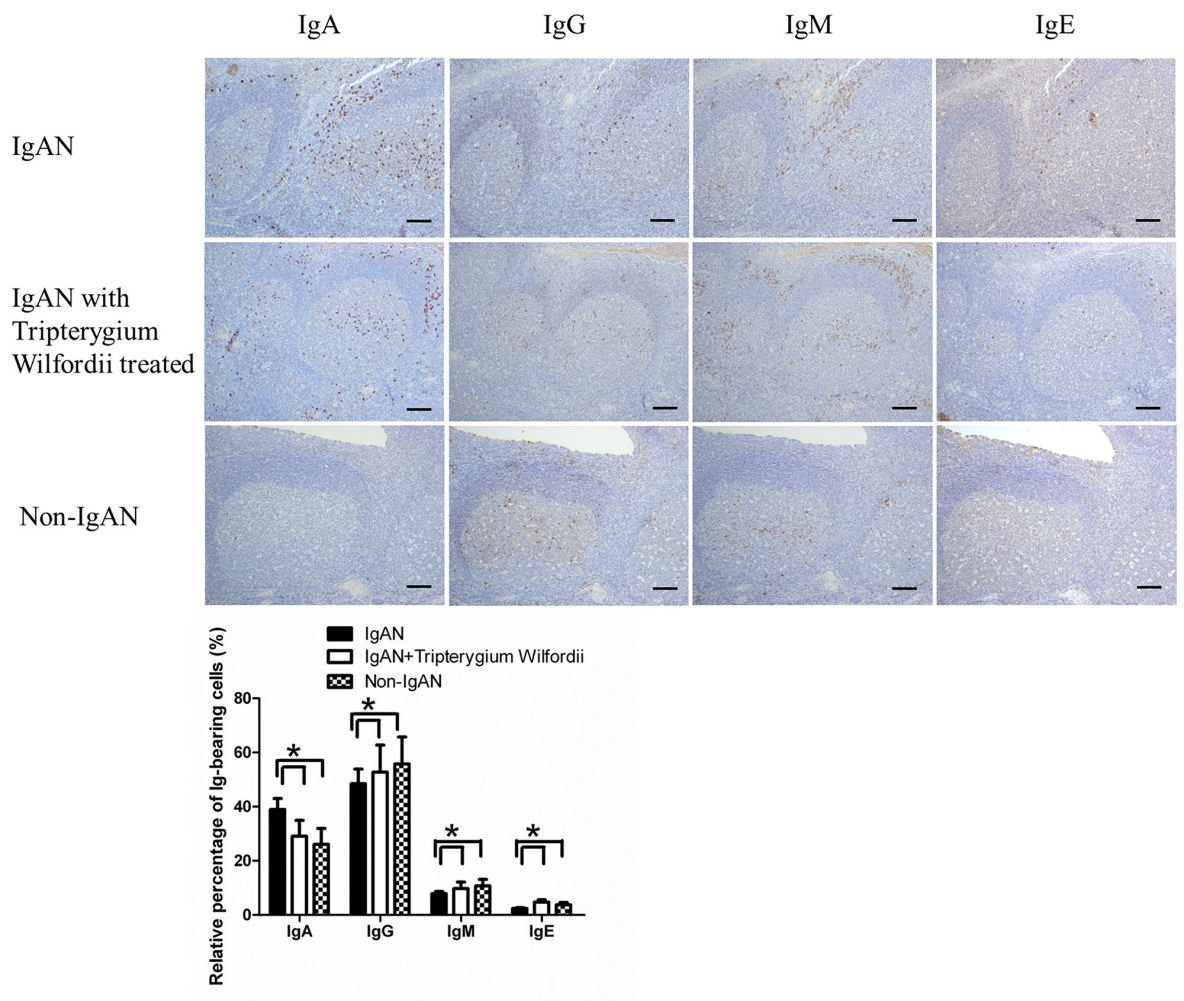
Among immunoglobulin classes, IgA was decreased in the tonsils of IgAN patients with Tripterygium Wilfordii treatment Immunohistochemistry on serial sections was used to show the expression of IgA, IgG, IgM and IgE in the tonsils. Bars, 500 μm. GC, germinal center. Positive cells were counted in low-power (100× magnification) fields for each patient. The slides were analyzed in blinded manner by two independent investigators. n = 20 for IgAN patients with Tripterygium Wilfordii treatment, n = 20 for IgAN patients without treatment and n = 20 for non-IgAN patients with chronic tonsillitis. Error bars indicate SEMs. ^*^, *P* < 0.05 (Mann-Whitney U test).

In IgAN patients with TW treatment, both the number (Figure 1) and relative percentage (Figure 2) of IgA-bearing cells were significantly decreased among all Ig-bearing cells. Meanwhile, the percentage of IgM-bearing cells was lower in IgAN patients without treatment compared to IgAN patients with Tripterygium Wilfordii treatment and non-IgAN chronic tonsillitis (Figure 2). The number of IgA1-bearing cells were significantly decreased in IgAN patients with TW treatment than in those without treatment (Supplementary Figure 2). We observed increased expression of TSLP in tonsillar GCs of IgAN patients compared to IgAN patients with TW treatment and non-IgAN chronic tonsillitis, and there was a positive correlation between IgA and TSLP expression levels in tonsils (R = 0.768, and P < 0.05 for Spearman’s correlation; Figure 1D).

### Decreased expression of TSLP, TSLPR, AID, and IgA-inducing cytokines in the tonsils of IgAN patients with TW treatment

**Figure 3:**
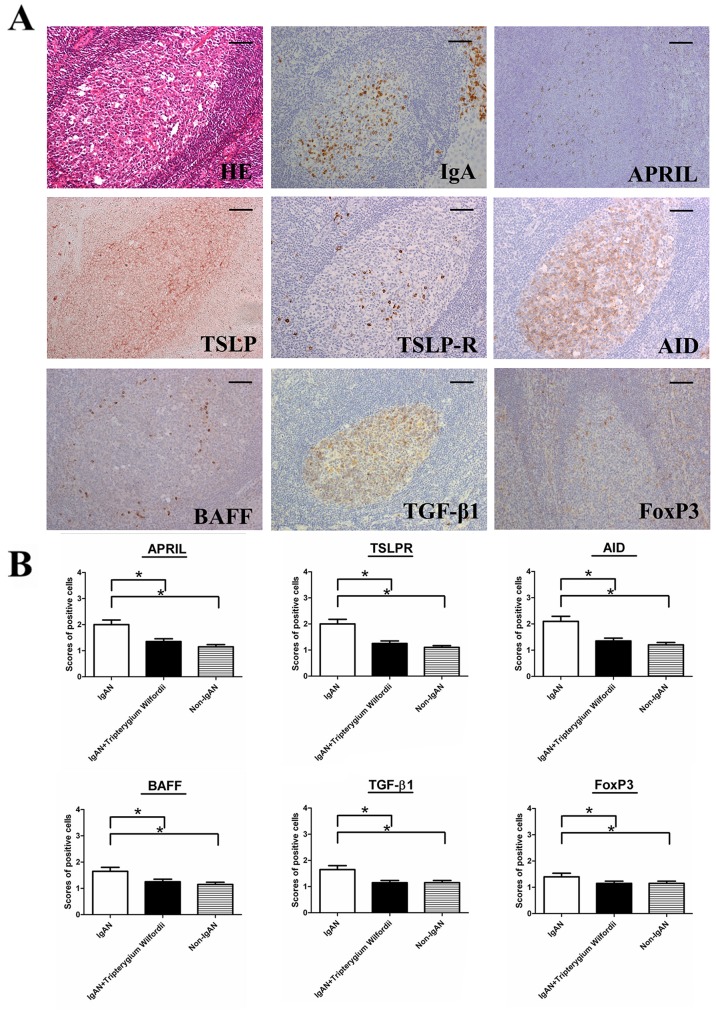
TSLP receptor (TSLPR), activation-induced cytidine deaminase (AID), transforming growth factor-β1 (TGF-β1), B cell-activating factor of the TNF family (BAFF) and a proliferation-inducing ligand (APRIL) were decreased in the tonsils of IgAN patients with Tripterygium Wilfordii treatment **(A)** Immunohistochemistry was used to demonstrate the expression of TSLPR, AID, and IgA-inducing cytokines (TGF-β1, BAFF, and APRIL in tonsillar germinal centers (GCs) of IgAN patients. Bars, 100 μm. **(B)** The scores of positive cells in the GCs were counted in 10 randomly chosen fields for each patient (400× magnification). The slides were analyzed in blinded manner by two independent investigators. n = 20 for IgAN patients with Tripterygium Wilfordii treatment, n = 20 for IgAN patients without treatment and n = 20 for non-IgAN patients with chronic tonsillitis. Error bars indicate SEMs. ^*^, *P* < 0.05; ^**^, *P* < 0.01 (Mann-Whitney U test).

The GC is the main site of IgA class switching in tonsils. To understand the mechanisms of IgA down regulation in the tonsils of IgAN patients, we assessed the expression of TSLP, TSLPR, AID, and IgA-inducing cytokines (i.e., TGF-β1, BAFF and APRIL) in the tonsillar GCs by IHC. Expression of AID, TGF-β1, BAFF and APRIL were decreased in the tonsils of IgAN patients with TW treatment than in those without treatment (Figure 3).

### Decreased expression of TSLP, TSLPR, AID, and IgA-inducing cytokines by FDCs in tonsillar GCs of IgAN patients with TW treatment

**Figure 4:**
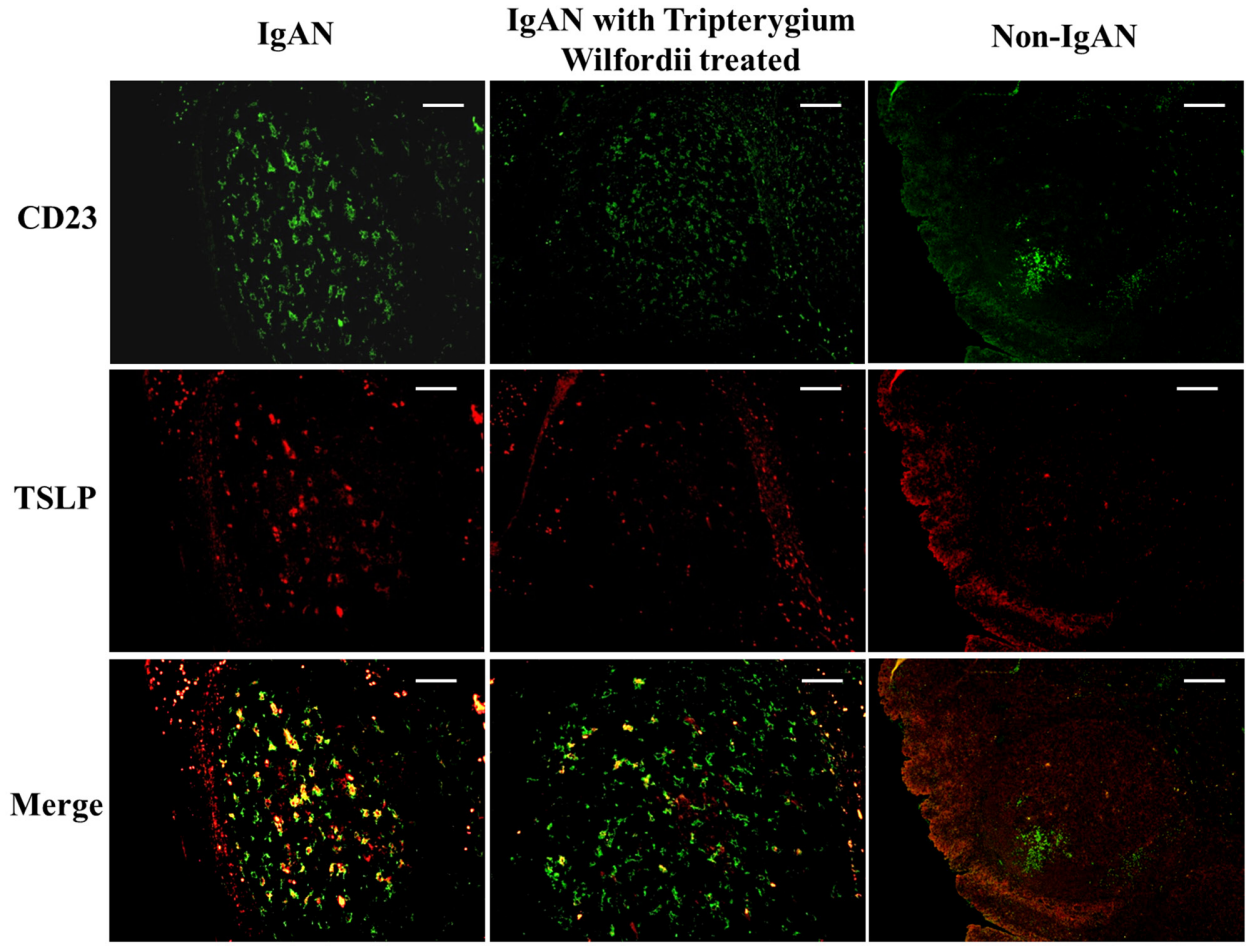
Thymic stromal lymphopoietin (TSLP) was co-expressed in follicular dendritic cells (FDCs) located within tonsillar germinal centers (GCs) Double immunofluorescence for TSLP (green) and CD23 (red) was performed in GCs. Bars, 100 μm. n = 20 for IgAN patients with Tripterygium Wilfordii treatment, n = 20 for IgAN patients without treatment and n = 20 for non-IgAN patients with chronic tonsillitis.

**Figure 5:**
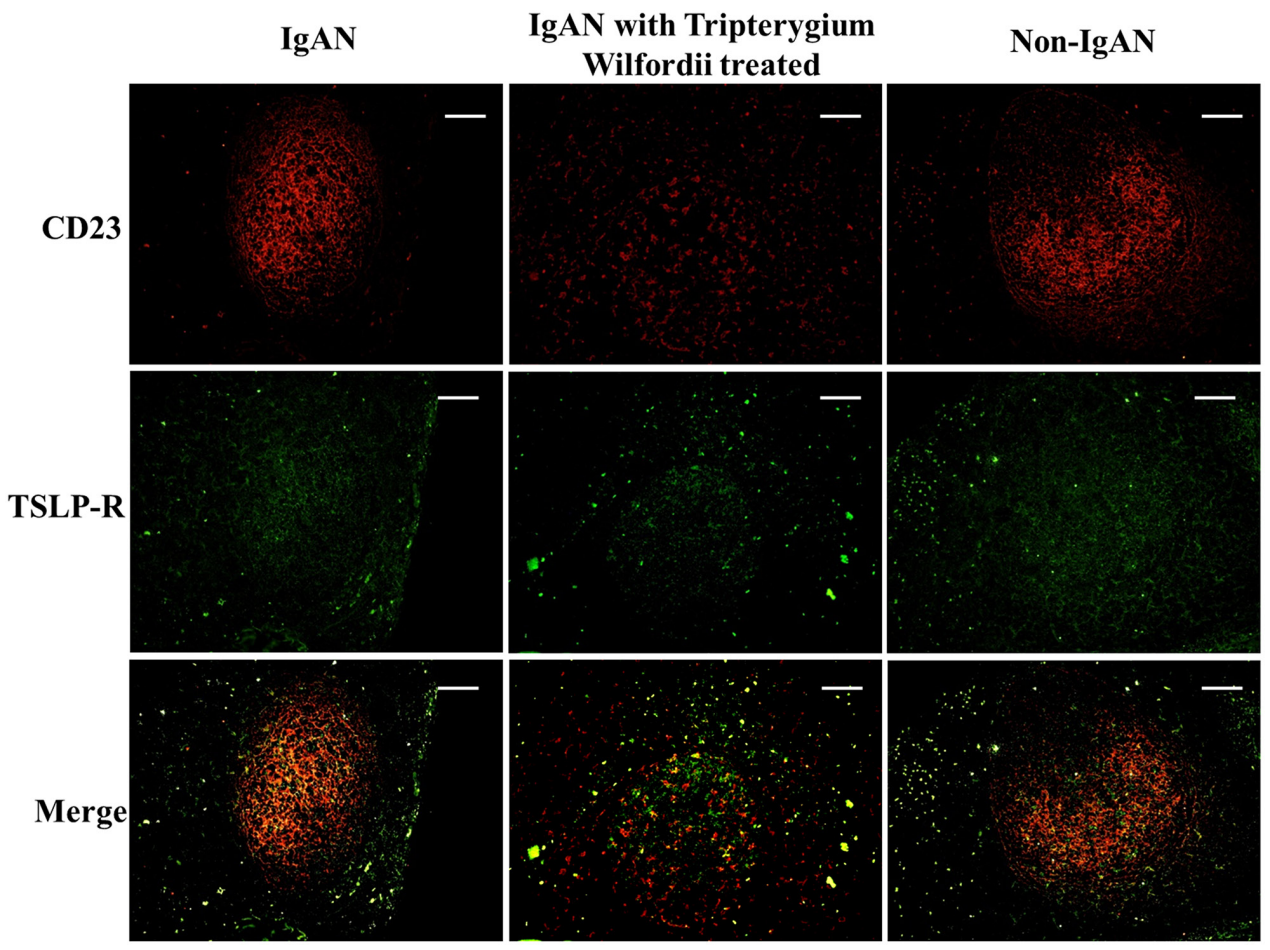
TSLP receptor (TSLPR) was co-expressed in FDCs located within tonsillar germinal centers (GCs) Double immunofluorescence for TSLPR (green) and CD23 (red) was performed in GCs. Bars, 100 μm. n = 20 for IgAN patients with Tripterygium Wilfordii treatment, n = 20 for IgAN patients without treatment and n = 20 for non-IgAN patients with chronic tonsillitis.

**Figure 6:**
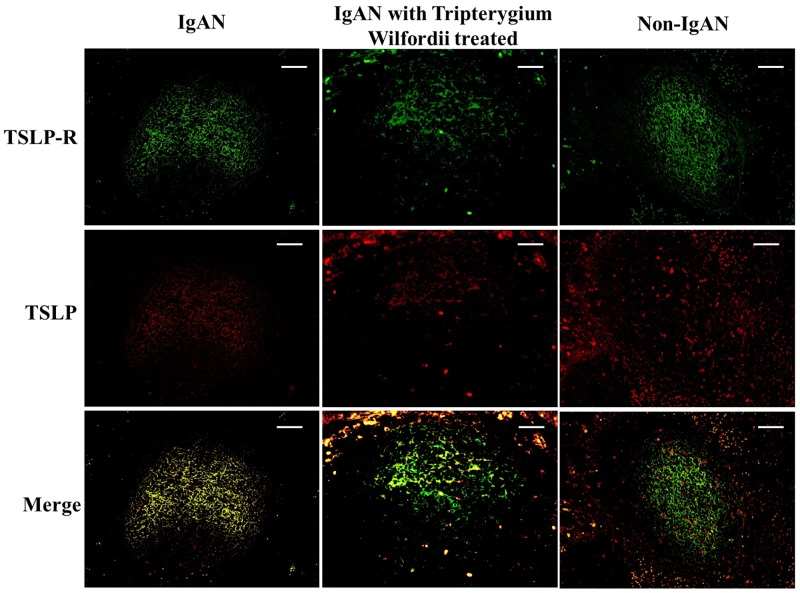
TSLP and TSLPR were co-expressed within tonsillar germinal centers (GCs) Double immunofluorescence for TSLP (red) and TSLPR (green) was performed in GCs. Bars, 100 μm. n = 20 for IgAN patients with Tripterygium Wilfordii treatment, n = 20 for IgAN patients without treatment and n = 20 for non-IgAN patients with chronic tonsillitis.

**Figure 7:**
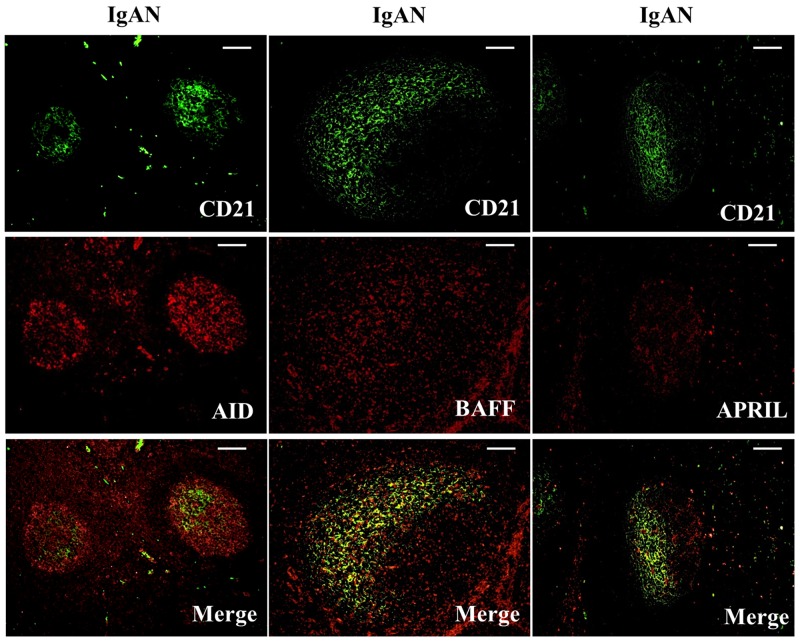
Activation-induced cytidine deaminase (AID), B cell-activating factor of the TNF family (BAFF) and a proliferation-inducing ligand (APRIL) were co-expressed in follicular dendritic cells (FDCs) located within tonsillar germinal centers (GCs) of IgAN patients Double immunofluorescence for AID (red) and CD21 (green); BAFF (red) and CD21 (green); and APRIL (red) and CD21 (green) was performed in GCs from IgAN patients. Bars, 100 μm. n = 20 for IgAN patients with Tripterygium Wilfordii treatment, n = 20 for IgAN patients without treatment and n = 20 for non-IgAN patients with chronic tonsillitis.

**Figure 8:**
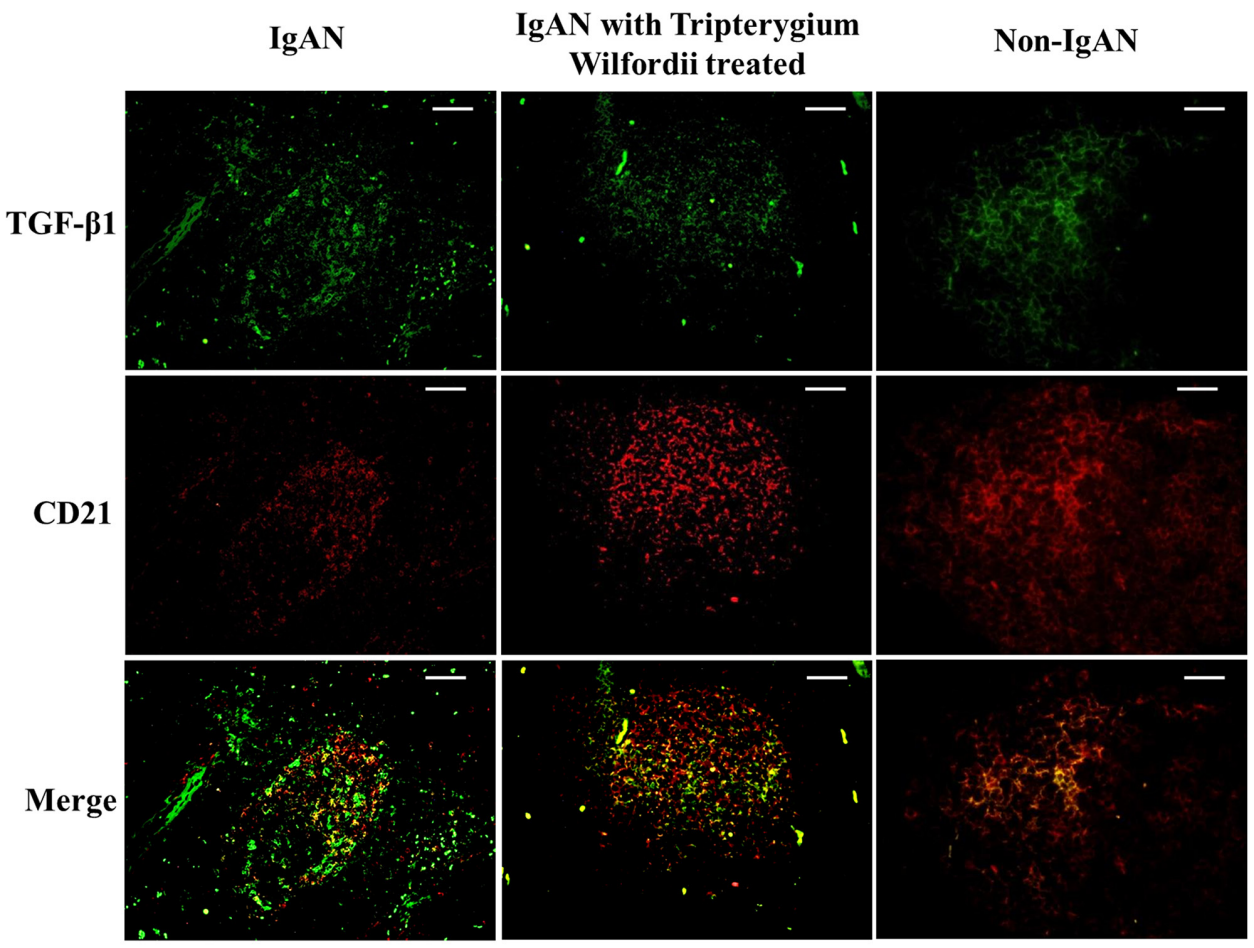
Transforming growth factor-β1 (TGF-β1) was co-expressed in FDCs located within tonsillar germinal centers (GCs) Double immunofluorescence for TGF-β1 (green) and CD21 (red) was performed in GCs. Bars, 100 μm. n = 20 for IgAN patients with Tripterygium Wilfordii treatment, n = 20 for IgAN patients without treatment and n = 20 for non-IgAN patients with chronic tonsillitis.

We examined whether tonsillar FDCs expressed AID and IgA-inducing cytokines using immunofluorescence double staining. Our results demonstrated that TSLP, TSLPR, AID, TGF-β1 and BAFF expression levels were dramatically decreased in tonsillar FDCs of IgAN patients with TW treatment as compared to IgAN patients without treatment (Figure 4-8).

### IgAN patients exhibited decreased expression of mRNAs encoding TSLP, TSLPR, AID, and IgA-inducing cytokines and reduced IgA class switching in GCs

**Figure 9:**
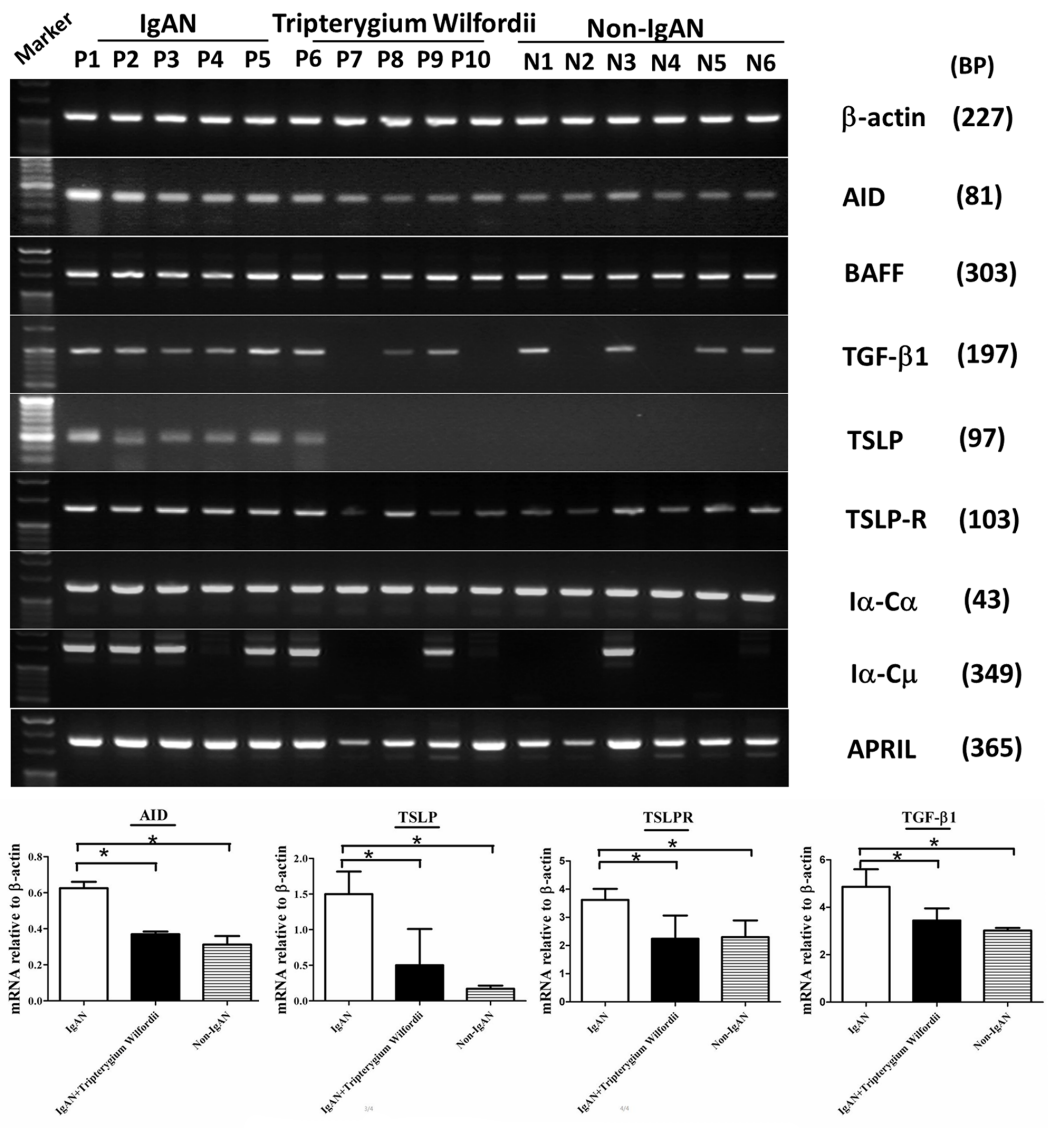
The mRNA expression levels of IgA class switching markers, thymic stromal lymphopoietin (TSLP), TSLP receptor (TSLPR), activation-induced cytidine deaminase (AID) and transforming growth factor-β1 (TGF-β1) were decreased in tonsillar GCs of IgAN patients with Tripterygium Wilfordii treatment RT-PCR was used to measure mRNAs encoding β-actin, TSLP, TSLPR, AID, TGF-β1, BAFF, APRIL, germline Cα (Iα-Cα), and switch circle (Iα-Cμ) in tonsillar GCs of IgAN patients and non-IgAN patients with chronic tonsillitis. *TSLP*, *TSLPR*, *AID*, and *TGF-β1* mRNA levels were determined by real-time PCR and normalized to β-actin mRNA levels in tonsillar GCs from IgAN patients with Tripterygium Wilfordii treatment (n = 20), IgAN patients without treatment (n = 20) and non-IgAN patients with chronic tonsillitis (n = 20). Error bars indicate SEMs. ^*^, *P* < 0.01 (Mann-Whitney U test).

To evaluate molecular changes in TSLP, TSLPR, AID, and IgA-inducing cytokines in tonsillar GCs of IgAN patients, tonsillar GCs enucleated by laser microdissection (Supplementary Figure 2) were used for reverse transcription polymerase chain reaction (RT-PCR) analysis (Figure 9). AID and Iα-Cα GLTs are indispensable for the initiation of CSR, [32] the expression of *Iα-Cμ* mRNA was detected in all IgAN patients and some controls. Moreover, *AID*, *Iα-Cα*, *TGF-β1*, *BAFF*, and *APRIL* mRNAs were detected in GCs from both the IgAN and non-IgAN groups by RT-PCR (Figure 9). Finally, we found and that *TSLP*, *TSLPR*, *AID*, and *TGF-β1* mRNA levels were decreased in GCs in the IgAN group with TW treatment compared with those without treatment (*P* < 0.05; Figure 9). More importantly, *TSLP* mRNA levels correlated with *AID* mRNA levels (R = 0.622, and *P* < 0.05 for Spearman’s correlation) and *TGF-β1* mRNA levels (R = 0.604, and *P* < 0.05).

### TW inhibit TSLP and IgA production in FDC-associated clusters

**Figure 10:**
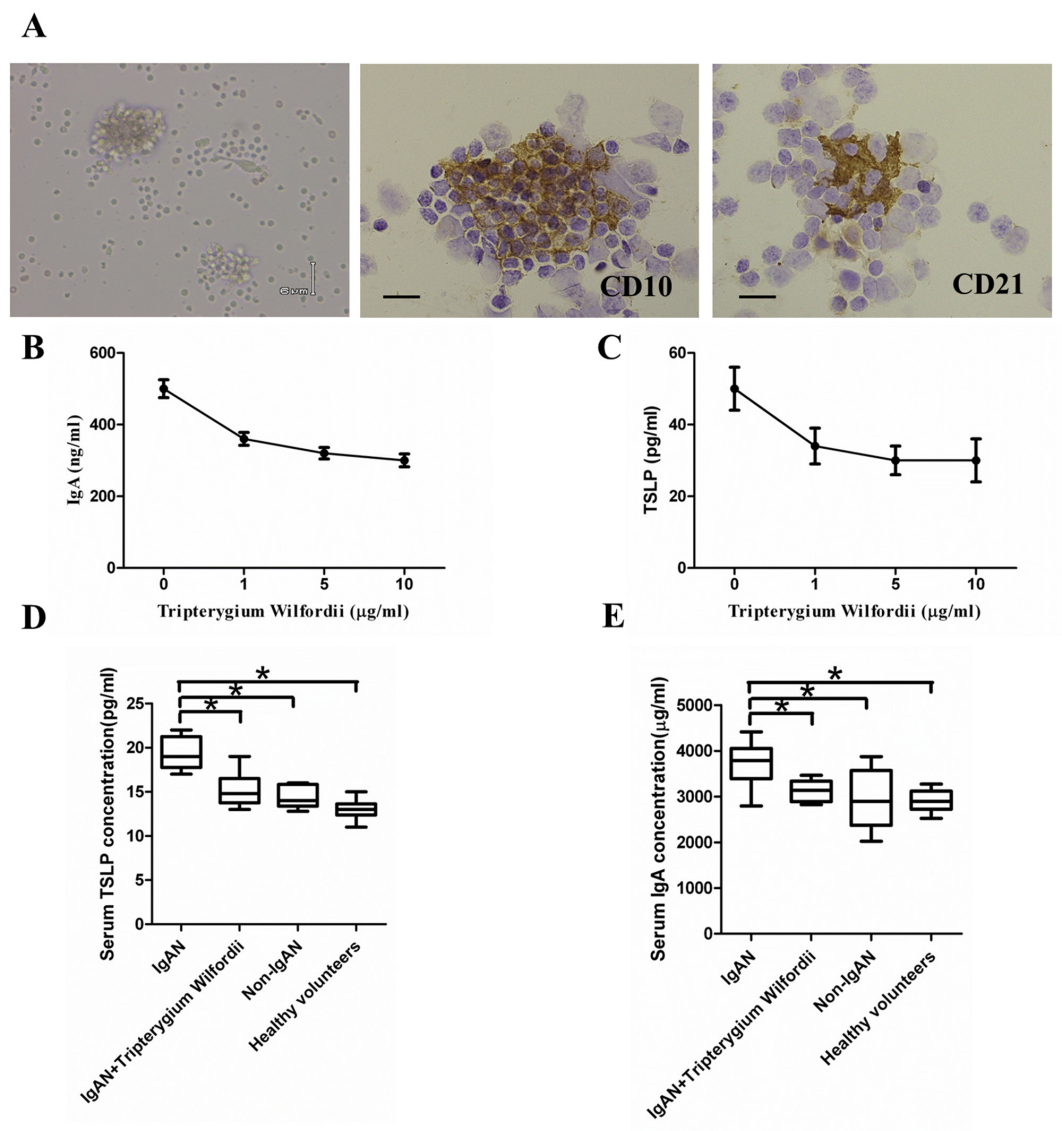
Thymic stromal lymphopoietin (TSLP) enhanced IgA production in follicular dendritic cell (FDC)-associated clusters through TSLP receptor (TSLPR), and serum IgA and TSLP concentrations **(A)** Left to right in the upper row: morphology, CD10^+^ GC cells and CD21^+^ FDCs in FDC-associated clusters isolated from tonsillar GCs of IgAN patients. FDC-associated clusters are composed of CD 10^+^ GC cells and CD21^+^ FDCs, with about 1 FDC per 10 lymphocytes in each FDC-associated cluster. **(B, C)** Left to right in the lower row: IgA and TSLP concentrations in the supernatants of FDC-associated clusters. IgA and TSLP were quantified in the supernatants using ELISA. Combined data (mean ± SD) from experiments using FDC-associated clusters from 3 IgAN patients are presented. **(D, E)** The IgA and TSLP concentrations in the sera of 20 IgAN patients with Tripterygium Wilfordii treatment, 20 IgAN patients without treatment, 20 non-IgAN patients with chronic tonsillitis patients, and 20 healthy volunteers were measured by ELISA. Error bars show means ± SEMs. ^*^, *P* < 0.05; ^**^, *P* < 0.01 (using the nonparametric Mann-Whitney U test).

FDC-associated clusters are composed of CD 10^+^ GC cells and CD21^+^ FDCs, with about 1 FDC per 10 lymphocytes in each FDC-associated cluster. To investigate the effects of TW on TSLP and IgA production, TW were added to FDC-associated clusters for 7 days. Interestingly, exposure to Tripterygium Wilfordii inhibit TSLP and IgA secretion in FDC-associated clusters (Figure 10).

### IgAN patients with TW treatment exhibited decreased serum IgA and TSLP as compared to those without treatment and nearly with normal controls

Serum IgA and TSLP concentrations were decreased in IgAN patients with TW treatment than in those without treatment, and nearly with normal controls (Figure 10). More importantly, the levels of serum TSLP correlated with the levels of serum IgA (R = 0.849, *P* < 0.05), IgA expression in GCs (R = 0.765, *P* < 0.05) and TSLP expression in GCs (R = 0.742, *P* < 0.05). The levels of serum IgA correlated with the degree of IgA expression in GCs (R = 0.765, *P* < 0.01) and TSLP expression in GCs (R = 0.672, *P* < 0.05). We also found IgAN patients with high mesangial score, segmental glomerulosclerosis, endocapillary hypercellularity, and tubular atrophy/interstitial fibrosis showed high serum IgA concentration (P <0.05) (Supplementary Figure 3).

## DISCUSSION

IgA nephropathy (IgAN) is characterized by a qualitative abnormality of IgA in the circulation and IgA deposition in the renal mesangium [15]. Mesangial deposition of IgA has been considered to be the initiating event in the pathogenesis of IgAN; Studies have suggested that IgA deposits in the glomerular mesangium may originate from the tonsils [16]. Recent studies have focused on the relationship between mucosal immunity and IgAN. Previous studies have indicated that tonsils in IgAN patients show reduced reticulization of the tonsillar crypt epithelium compared with those of non-IgAN chronic tonsillitis. Several bacteria specific to the tonsils has known to correlate with the prognosis of IgAN [17, 18]. These characteristics may induce unusual immune responses in tonsils, which may underlie the pathogenesis of IgAN. It has been found that TW acquire good benefits in IgAN. These findings suggest that TW may be related to the IgA production in the tonsils. However, the mechanisms through which TW improves renal outcomes are unclear. TW usually used to target tonsillitis, while little is known about the involvement of TW in IgA production in the tonsils.

Palatine tonsils, the major component of nasopharynx-associated lymphoid tissue, play a major role in mucosal immunity in human airways [19]. Tonsillar GCs act as important inductive sites for mucosal B cell responses, [20] supported by FDCs and a small number of follicular helper T cells. Once activated, follicular naive IgM^+^IgD^-^ B cells may undergo IgA class switching and then differentiate into IgA^+^ plasma cells or may then migrate to systemic sites [21].

In the present study, we found that both the number and percentage of IgA-bearing cells were significantly decreased among immunoglobulins in IgAN patients with TW treatment (Figure 1-2). Additionally, we observed a correlation between tonsillar IgA expression and serum IgA concentrations in patients with IgAN. Therefore, TW may be involved in IgA production in the tonsils of IgAN patients, resulting in the benefits of IgAN.

Newly emerging B cells generally express IgM antibodies, but these antibodies can undergo class switching into other isotypes upon stimulation by antigen. As described in the Introduction, together with Iα-Cα GLTs, Iα-Cμ switch circles have short half-lives, and detection of these targets therefore indicates ongoing CSR [21]. In our study, the percentage of IgA^+^ cells was significantly decreased, while that of IgM^+^ cells was increased in IgAN patients with TW treatment compared with those without treatment, similar to Non-IgAN. In addition, the expression levels of Ia-Cμ mRNA were decreased in IgAN patients with TW treatment compared with those without treatment, indicating that IgA class switching has been inhibited in the tonsils of IgAN patients with TW treatment.

A number of proteins are important for CSR. AID is the crucial protein promotes DNA double-strand breaks, an essential mechanism of CSR [10, 21]. Furthermore, TGF-β1 and two other TNF-family cytokines (BAFF and APRIL) have been shown to have important roles in B-cell biology and to contribute to IgA production, affecting the augmentation of B-cell antigen presentation, costimulation of B-cell activation, enhancement of B-cell survival, and differentiation of recently switched B cells into plasmablasts [22]. The expression of AID, TGF-β1, BAFF and APRIL protein (Figure 3A-3B) and mRNA (Figure 8A, 8D) in GCs was decreased in IgAN patients with TW treatment compared to those without treatment, corresponding to the inhibited IgA class switching observed in the tonsils of IgAN patients. Decreased expression of Foxp3 may be associated with immunosuppression effect of TW in GCs by hampering GC development in the tonsils of patients with TW treatment. Thus, these data suggest that AID, TGF-β1, BAFF and APRIL may be involved in IgA production within tonsils of IgAN patients with TW treatment.

A previous study demonstrated that the tonsillar crypt epithelium can be activated to secrete TSLP, a cytokine that further promotes CSR [23, 24]. TSLP released by intestinal epithelial cells has been shown to enhance the production of APRIL via stimulation of intestinal myeloid DCs to induce IgA production by B cells [25]. It has been described that TSLP affects the early B-cell progenitor stages to promote B-cell differentiation into mature B cells. Our previous study has indicated that TSLP overexpression in tonsillar FDCs may promoted IgA class switching in IgAN patients through the cooperative roles of AID, TGF-β1, BAFF, and APRIL, and the critical role of the TSLP-TSLPR autocrine/paracrine loop in FDCs on IgA class switching [7]. Similarly, in our data, we observed decreased expression of TSLP in tonsillar GCs of IgAN with TW treatment (Figure 3A-3B), which correlated with the expression of IgA (Figure 4).

Based on the present and previous data, a possible model was proposed whereby TW induce the decreased expression of AID, TGF-β1, BAFF and APRIL by tonsillar FDCs depends on signaling through pathways involving TSLP and TSLPR, inhibiting the generation of IgA^+^ B cells and IgA^+^ plasmablasts.

TSLP levels have been reported to be increased in the sera of patients with IgAN, women with endometriosis and children with atopic dermatitis, as well as the synovial fluid of patients with rheumatoid arthritis, indicating its role in allergic and non-allergic inflammation [26, 27]. In this study, Serum IgA and TSLP concentrations were decreased in IgAN patients with TW treatment than in those without treatment, and nearly with normal controls. Serum TSLP also correlated with TSLP and IgA expression in the tonsils and with the degree of serum IgA. These data suggest that interactions among serum TSLP, tonsillar TSLP, tonsillar IgA production, and serum IgA may be responsible for the mechanisms underlying the role of TW in the treatment of IgAN.

## MATERIALS AND METHODS

### Patients and sera

Palatine tonsils and sera were obtained from 30 patients with biopsy-proven IgAN (age 16-73 years, mean 48.4) with TW treatment before tonsillity, 30 patients with biopsy-proven IgAN (age 16-73 years, mean 42.4) without treatment and 30 patients with chronic tonsillitis but lacking renal diseases and history of hematuria following tonsillectomy (age 22-59 years, mean 36.0). IgAN patients with TW treatment received 60mg/d for 60 days of dosing before tonsillity. All renal samples were diagnosed according to the Oxford classification by two well-trained renal pathologists. The 4 pathologic variables of the Oxford classification were scored as follows: mesangial score less than or equal to 0.5 (M0) or greater than 0.5 (M1), segmental glomerulosclerosis absent (S0) or present (Supplementary Figure 1), endocapillary hypercellularity absent (E0) or present (E1), and tubular atrophy/interstitial fibrosis less than or equal to 25% (T0), 26%–50% (T1), or more than 50% (T2). Indications for tonsillectomy for IgAN were demonstrated in previous studies as patients with hematuria-type IgAN, especially those presenting hematuria after tonsillar infection; with a baseline creatinine level of ≤ 2mg/dl. Patients were recruited at Harbin Medical University Cancer Hospital (Harbin, China) and the First Affiliated Hospital of Hei Longjiang University of Chinese Medicine (Harbin, China). Cases with Henoch-Schonlein purpura, palmoplantar pustulosis, rheumatic arthritis and ossification, liver cirrhosis, systemic lupus erythematosus, or other systemic diseases were excluded. Sera from 20 healthy sex- and age-matched volunteers (age 28-66 years, mean 40.1) with negative urinalysis results were collected as normal controls. The clinical parameters for patients and volunteers were collected (Table 1). This study was conducted in accordance with the Declaration of Helsinki, and written informed consent obtained from each participant. Approval for this study was obtained from the Medical Ethics Committees of First Affiliated Hospital of Hei Longjiang University of Chinese Medicine (HZYLLBA201714).

**Table 1:** Profiles and clinical parameters of patients

Parameter	IgAN group without TW tratment (n =30)	IgAN group before TW tratment (n =30)	IgAN group after TW tratment (n =30)	Chronic tonsillitis group (n = 30)	Healthy volunteer group (n = 20)	Normal value (reference)
Age (years)	42.37 ± 14.1	48.36 ± 16.1	48.36 ± 16.1	36 ± 11.2	40.1 ± 12.14	-
Gender (male/female)	15/15	15/15	15/15	16/14	6/4	-
Urinary protein (mg/day)	0.155 ± 0.35	0.105 ± 0.65	0.10 ± 0.26	N.D.	N.D.	0–0.15
Hematuria (/hpf)	42.07 ± 33.54	33.07 ± 33.54	13.04 ± 14.60	0	0	0
Serum creatinine (mg/dL)	0.92 ± 0.28	0.82 ± 0.48	0.65 ± 0.22	N.D.	N.D.	0.47–0.79
Uric acid (mg/dL)	5.83 ± 1.22	5.46 ± 1.46	4.65 ± 1.32	N.D.	N.D.	2.4–5.6
CRP (mg/dL)	0.87 ± 0.94	0.46 ± 0.65	0.28 ± 0.4	N.D.	N.D.	0–0.24
Serum complement (U/mL)	47.68 ± 8.42	43.68 ± 6.23	30.15 ± 4.22	N.D.	N.D.	28–44

Note: values are the means ± SDs. hpf = high-power field; N.D. = no data

### Isolation and identification of FDC-associated clusters from tonsillar GCs

FDC-associated clusters from tonsillar GCs of IgAN patients were isolated as described previously [26, 27]. Briefly, surgically removed fresh palatine tonsils were cut into 300-μm-thick slices using a microslicer (DTK-1000, Dosaka EM, Co. Ltd., Kyoto, Japan) and floated in cold phosphate-buffered saline (PBS) containing 0.4% bovine serum albumin (BSA; Sigma, St. Louis, MO, USA). Approximately 200 GCs were enucleated from each tonsil with an ophthalmic V-Lance Knife (Alcon Surgical, Fort Worth, TX, USA) under a stereo microscope. The isolated GCs were digested for 20 min at 37°C in PBS with 0.05% collagenase (Type II, Gibco, Grand Island, NY, USA), 0.05% dispase (Grade 1), and 0.004% DNase (Sigma). The fraction of freed cells was collected in cold PBS containing 0.4% BSA, purified by repetitive 1G sedimentation, and filtered through nylon-wool. To remove macrophages, the FDC-cluster-rich fraction was cultured in a plastic culture dish with RPM1 1640 medium (Sigma) containing 10% fetal bovine serum (FBS; Gibco) at 37°C in 5% CO_2_ for 60 min. Nonadherent cells were transferred on plastic covers to 24-well culture plates (Falcon, Becton Dickinson and Company, Franklin Lakes, NJ, USA) and maintained in these same conditions. After 6 h, the plastic covers were briefly washed and transferred into wells with fresh medium (RPMI 1640, 2 mM l-glutamine, 50 μM 2-mercaptoethanol, 100 U/mL penicillin, 100 μg/mL streptomycin, and 10% FBS) for further assay. IHC for CD10 (56C6; mouse IgG1, Nichirei, Tokyo, Japan) and CD21 (1F8; mouse IgG1 κ, Dako, Glostrup, Denmark) was performed to characterize the cells within the clusters.

### Antibodies, IHC, and immunofluorescence

Primary and secondary antibodies used for IHC and immunofluorescence were shown in Table 2. For IHC, formalin-fixed, paraffin-embedded tonsil sections (4 μm in thickness) were blocked with 1% H_2_O_2_ and then subjected to antigen retrieval in trypsin for 30 min at 37°C; EDTA (pH 9.0; Maixin Biotechnologies) for 20 min at 120°C in an autoclave; or immunosaver (pH 7.4; Nisshin EM, Tokyo, Japan) for 45 min at 98°C in an electric pot. IHC was performed using either the streptavidin-biotin-peroxidase complex (strept-ABC) or the alkaline phosphatase anti-alkaline phosphatase (APAAP) method as previously reported [28]. Sections were visualized using 3,3’-diaminobenzidine (DAB; Maixin Biotechnologies) or 3-amino-9-ethylcarbazole (AEC; Maixin Biotechnologies). Specific isotype control antibodies and PBS (omitting primary antibodies) were used as negative controls. Slides were visualized on a microscope (BX45; Olympus, Tokyo, Japan) using a digital camera (DP70; Olympus). The number of positive cells for IHC and immunofluorescence were scored as 0 (absent), 1+ (< 25% of GC cells), 2+ (25%-50% of GC cells), 3+ (50%-75% of GC cells), or 4+ (> 75% of GC cells).

**Table 2:** Antibodies for immunohistochemistry and immunofluorescence

Antibodies (Clone, Isotype)	Dilution	Antigen retrieval	Source
Rabbit anti-IgA	Ready-to-use	Trypsin	Nichirei
Mouse anti-IgG (A57H, IgM)	Ready-to-use	Trypsin	Nichirei
Rabbit anti-IgE	× 100	Trypsin	Dako
rabbit anti-IgM	× 60	Trypsin	Covance
Rabbit anti-human TSLP (ab47943, IgG)	10 μg/ml	Immunosaver	Abcam
Goat anti-human TSLPR (IgG)	10 μg/ml	Immunosaver	R&D
Rat anti-human AID (EK2 5G9, IgG2b)	× 100	Immunosaver	Cell Signaling
Goat anti-human TGF-β1 (IgG)	10 μg/ml	Immunosaver	R&D
Rat anti-human BAFF (ab16081, IgG2b)	× 100	Immunosaver	Abcam
Mouse anti-human APRIL (IgG1)	× 100	Immunosaver	Enzo
Mouse anti-CD10 (56C6, IgG1)	Ready-to-use	EDTA	Nichirei
Mouse anti-CD21 (1F8, IgG1)	× 40	Immunosaver	Dako
Mouse anti-CD23 (1B12, IgG1)	× 30	Immunosaver	Novocastra
HRP-donkey F (ab´)2 anti-goat IgG	× 100	Not use	Abcam
Goat anti-rabbit secondary antibody	× 100	Not use	Jackson
Biotinylated anti-sheep IgG	× 100	Not use	Vector
Rhodamine-donkey anti-mouse IgG	× 100	Not use	Jackson
FITC-donkey anti-mouse IgG	× 100	Not use	Jackson
FITC-goat anti-rabbit IgG	× 100	Not use	Jackson
FITC-donkey anti-goat IgG	× 100	Not use	Beckman Coulter
Alexa Fluor 568-goat anti-rat IgG	× 100	Not use	Invitrogen
Fluor 555-goat anti-rabbit IgG	× 100	Not use	Invitrogen

For IHC, FDC-associated clusters were placed onto Millicell EZ 4-well glass slides (EMD Millipore Corporation, Billerica, MA, USA) and cultured in RPM1 1640 containing 10% FBS before staining. FDC-associated clusters cultured on glass slides were rinsed in PBS and fixed with 4% paraformaldehyde in PBS for 20 min at room temperature. Slides were then subjected to IHC as described above, excluding the dewaxing and antigen retrieval steps.

Multiple immunofluorescence labeling of formalin-fixed, paraffin-embedded sections was performed as previously described [29]. Briefly, dewaxing and antigen retrieval were performed using immunosaver (pH 7.4; Nisshin EM) for 45 min at 98°C. Sections were washed in PBS and rinsed in PBS containing 1% BSA and 2% fetal calf serum. Sections were then incubated with primary antibodies overnight at 4°C followed by incubation with other primary antibodies for 1-2 h at room temperature. Sections were washed in PBS between each step. The primary antibody incubation was followed by incubation with fluorochrome-conjugated secondary antibodies. There was no cross reactivity of the antibodies. Slides were mounted with Fluoromount (Diagnostic BioSystems, Pleasanton, CA, USA) and analyzed under a microscope (BX53; Olympus) using a BX3-URA fluorescence system (Olympus). Multiple immunofluorescence labeling ofFDC-associated clusters was performed as described above, excluding the dewaxing and antigen retrieval steps.

### Laser-capture microdissection (LCM) of tonsillar GCs, RNA extraction and RT-PCR analysis

LCM was performed as previously described, [30] with minor modifications. Surgically removed fresh palatine tonsils were fixed with RNAlater RNA Stabilization Reagent (abcam) for 12 h at 4°C, embedded in optimal cutting temperature compound (Sakura Tissue-Tek 4583; Sakura Finetek USA, Inc., Torrance, CA, USA), and cut into 8-μm-thick sections on a freezing microtome. LCM was performed using a PALM Microlaser System (PALM Microlaser Technologies AG, Bernried, Germany) according to published procedures. Thirty sections for each tonsil were placed on cooled PEN Membrane Glass Slides (LCM0522, Applied Biosystems, Carlsbad, CA, USA). GCs were easily identifiable at low magnification on hematoxylin and eosin-stained sections. Two thousand GC components for each tonsil were captured and collected into a 0.5-mL RNase-free microcentrifuge tube (PALM Microlaser Technologies AG) containing RNAlater and immediately used for RNA extraction or frozen at -80°C until RNA extraction.

Total RNA was extracted and purified from LCM-captured cells using an RNeasy Micro kit (Qiagen, Hilden, Germany), including a DNase treatment step, according to the manufacturer’s instructions. Complementary DNA (cDNA) was synthesized using a QuantiTect Reverse Transcription Kit (Qiagen). The resulting cDNA was used as a template for PCR analysis. The forward- and reverse-specific primers, amplicon sizes, and annealing temperatures were as follows: β-actin 5′-CAGAGCAAGAGAGGCATCCT-3′ (forward) and 5′-ACGTACATGGCTGGGGTG-3′ (reverse), 227 bp, 55°C; TSLP 5′-TATGAGTGGGACCAAAAGTACCG-3′ (forward) and 5′-GGGATTGAAGGTTAGGCTCTGG-3′ (reverse), 97 bp, 55°C; TSLPR 5′-GAGTGGCAGTCCAAACAGGAA-3′ (forward) and 5′-ACATCCTCCATAGCCTTCACC-3′ (reverse), 103 bp, 62°C; IL-7Rα 5′-TGGACGCATGTGAATTTATC-3′ (forward) and 5′-CATTCACTCCAGAAGCCTTT-3′ (reverse), 130 bp, 57°C; AID 5′-TCGGCGTGAGACCTACC-3′ (forward) and 5′-CGAAGATAACCAAAGTCCAGTG-3′ (reverse), 81 bp, 56°C; TGF-β1 5′-ACCAACTATTGCTTCAGCTC-3′ (forward) and 5′-TTATGCTGGTTGTACAGGG-3′ (reverse), 197 bp, 50°C; BAFF 5′-ACCGCGGGACTGAAAATCT-3′ (forward) and 5′-TCCCATGGCGTAGGTCTTATC-3′ (reverse), 303 bp, 60°C; APRIL 5′-GCTCATGCCAGCCTCATCTC-3′ (forward) and 5′-CCAGGTGCAGGACAGAGTGCT-3′ (reverse), 365 bp, 67°C; and germline Iα-Cα mRNA 5′-CCAAGGTCTTCCCGCTGAG-3′ (forward) and 5′-CCATCTGGCTGGGTGCTG-3′ (reverse), 43 bp, 56°C. For nested PCR was for switch circle Iα-Cμ mRNA, primers and temperatures were as follows: forward primer for first round, 5′-CACAGCCAGCGAGGCAGAGC-3′; reverse primer for first round, 5′-ACGAAGACGCTCACTTTGGG-3′; annealing temperature for first round, 51°C; forward primer for second round, 5′-TGAGTGGACCTGCCATGA-3′; reverse primer for second round, 5′-CGTCTGTGCCTGCATGACG-3′; amplicon length, 349 bp; annealing temperature for second round, 58°C. PCR products were subjected to 4% agarose gel electrophoresis and visualized by ethidium bromide.

### Quantitative real-time PCR analysis

Equal amounts of RNA (50 ng) from samples were reverse transcribed using a QuantiTect RT kit (Qiagen). Resulting cDNA was amplified with a Fast SYBR Green Master Mix (Applied Biosystems) according to the manufacturer’s instructions, and samples were subjected to PCR on a 7500 Fast Real-Time PCR System (Applied Biosystems). Primers for TSLP, TSLPR, and AID were as described in the RT-PCR section. Additional primers included TGF-β1 forward (5′-GTGTGGAGCAACATGTGGAACTCTA-3′), and TGF-β1 reverse (5′-TTGGTTCAGCCACTGCCGTA-3′). Relative expression was determined using the relative standard curve method. Data were normalized to β-actin expression.

### Preparation of TW

TW were extracted by the First Affiliated Hospital of Hei Longjiang University of Chinese Medicine as described previously. Briefly, the debarked roots of Tripterygium Wilfordii were grounded (∼100 g) and extracted with adequate volumes of ethanol (95%, 1.8 L) three times (0.6 L each time) after overnight maceration followed by sonication for 30 min. The solvent from the fltered extract was evaporated (Buchi Rotary Evaporator R200 System, BUCHI Corp., New Castle, DE, USA). The dry residue so obtained was fractionated by adsorbing on silica gel 60 and sequentially extracted with hexane, chloroform, acetone and ethanol (95%) to obtain fractions of increasing polarity. The ethanol extract was subsequently selected for further study, since it is less toxic to the cells and is the preferred solvent for usual herbal product manufacturing.

The use of CE as a biological assay was utilized for the quality measurement of our TW extract (in conformance to “FDA Guidance on Botanical Drug Development”, FDA, 2015). Similar CE values (CV<30%) have been observed for our TW extract when tested at different times, indicating our TW extract method can yield a relatively consistent and stable value throughout the study period.

### Cell culture to assess IgA production

For IgA production analysis in FDC-associated clusters, the base cell culture media was supplemented with TW at 1 μg/mL, 5 μg/mL, or 10 μg/mL for the initial 7 days of culture. After collection of supernatants, qualitative detection of TSLP and IgA in cell supernatants was performed using Human TSLP ELISA MAX Deluxe Sets (Biolegend, San Diego, CA, USA) and IgA Human ELISA Kit (ab137980; Abcam).

### Measurement of TSLP and IgA in serum

Serum levels of TSLP and IgA were measured using Human TSLP ELISA MAX Deluxe Sets (Biolegend, San Diego, CA, USA) and an IgA Human ELISA Kit (ab137980; Abcam) according to the manufacturers’ protocols. The absorbance was read at 450 and 550 nm using a Varioskan Flash 2.4 System (Thermo Fisher scientific, Waltham, MA, USA). The sensitivity of the assay was 2 pg/mL for TSLP and 1.5 ng/mL for IgA.

### Statistical analysis

Statistical analyses were performed with the Mann-Whitney *U* test, Spearman’s correlation analysis (SAS Institute Inc., Cary, NC, USA) as indicated in details in figure legends. Differences with *p*-values of less than 0.05 were considered significant.

## CONCLUSION

This study demonstrated that the expression of TSLP and IgA inducing cytokines were decreased in the tonsils of IgAN patients with TW treatment compared with those without treatment, followed by significantly decreased of IgA-bearing cells. Therefore, Inhibition of IgA production by IgA class switching and interactions in the tonsils may explain the favorable outcome of TW in IgAN patients with elevated serum IgA. This is the first report to demonstrate that TW may be involved in IgA production in the tonsils of IgAN patients, by inhibiting IgA class switching in IgAN patients through the cooperative roles of AID, TGF-β1, BAFF, and APRIL, which may represent a promising strategy for therapeutic intervention in IgAN.

## SUPPLEMENTARY MATERIALS FIGURES



## Abbreviations

AEC: 3-amino-9-ethylcarbazole
AID: activation-induced cytidine deaminase
APRIL: a proliferation-inducing ligand
BAFF: B cell-activating factor of the TNF family
BSA: bovine serum albumin
cDNA: complementary DNA
CSR: class switch recombination
DAB: 3,3’-diaminobenzidine
DCs: dendritic cells
FBS: fetal bovine serum
FDCs: follicular dendritic cells
GC: germinal center
GLTs: germline transcripts
HRP: horseradish peroxidase
IgAN: IgA nephropathy
IHC: immunohistochemistry
LCM: laser-capture microdissection
PBS: phosphate-buffered saline
RT-PCR: reverse transcription polymerase chain reaction
TGF-β1: transforming growth factor-β1
TNF: tumor necrosis factor
TSLP: thymic stromal lymphopoietin
TSLPR: TSLP receptor
TW: Tripterygium Wilfordii
