# Supplementary material for: Tripterygium Wilfordii inhibits tonsillar IgA production by downregulating IgA class switching in IgA nephropathy
Source: Oncotarget. 2017 Nov 20;8(65):109027–42. doi: 10.18632/oncotarget.22561 (PMC5752501; doi:10.18632/oncotarget.22561)
Supplement: Supplementary file 1 [file oncotarget-08-109027-s001.pdf]

Tripterygium Wilfordii inhibits tonsillar IgA production by downregulating IgA class switching in IgA nephropathy

SUPPLEMENTARY MATERIALS

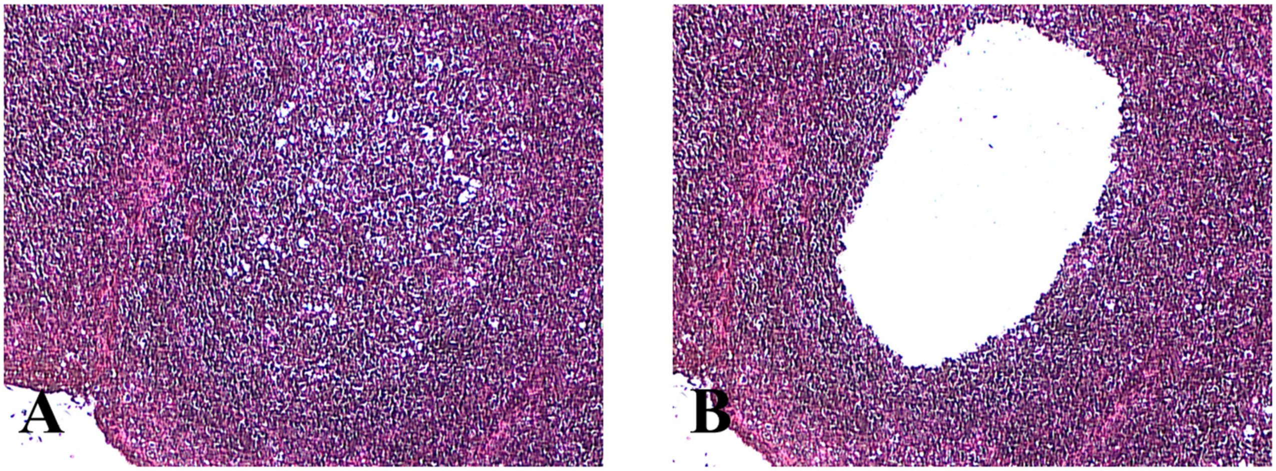

Supplementary Figure 1: Laser capture microdissection was performed to extract GC components from tonsils. HE staining of a sample before **(A)** and after **(B)** laser capture microdissection of the GC components in tonsils fixed by periodate-lysine-paraformaldehyde (PLP) and RNAlater.

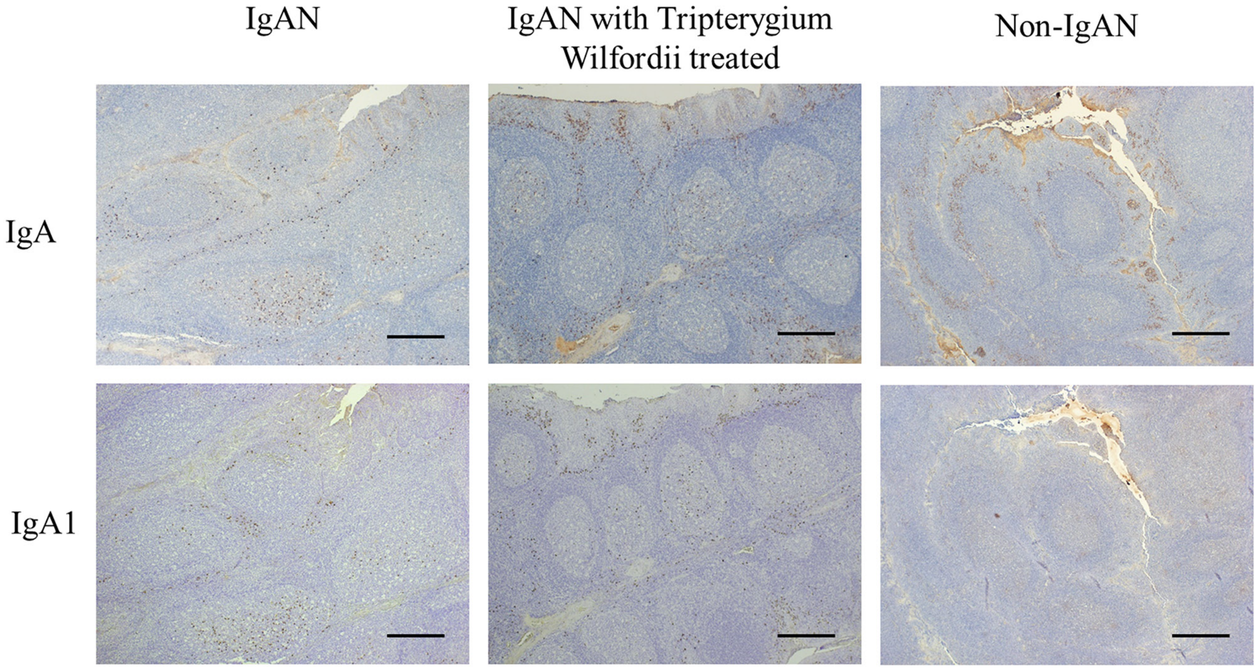

Supplementary Figure 2: IgAN patients with Tripterygium Wilfordii treatment exhibited decreased numbers of IgA1-bearing cells in their tonsils. Immunohistochemistry of IgA1 in the tonsils of IgAN patients and non-IgAN patients with chronic tonsillitis showed IgA1-bearing cells in the follicular germinal centers (GCs), reticular crypt epithelium (Ep), and subepithelial area. Bars, 200 μm.

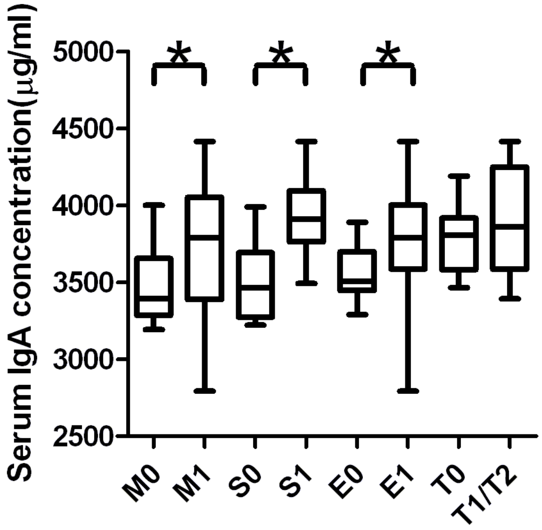

Supplementary Figure 3: Relationship between histopathological parameters and serum IgA level of IgAN.
